# Editorial: Cell-to-cell communications in alcohol-associated, metabolic-related and viral liver diseases

**DOI:** 10.3389/fphys.2023.1269042

**Published:** 2023-10-10

**Authors:** Natalia A. Osna, Kenneth E. Sherman, Pranoti Mandrekar, Kusum K. Kharbanda

**Affiliations:** ^1^ Research Service, Veterans Affairs Nebraska-Western Iowa Health Care System, Omaha, NE, United States; ^2^ Department of Medicine, University of Nebraska Medical Center, Omaha, NE, United States; ^3^ Department of Pharmacology and Experimental Neuroscience, University of Nebraska Medical Center, Omaha, NE, United States; ^4^ Division of Digestive Diseases, Department of Medicine, University of Cincinnati College of Medicine, Cincinnati, OH, United States; ^5^ Gastroenterology Division, Department of Medicine, Massachusetts General Hospital, Boston, MA, United States; ^6^ Department of Medicine, University of Massachusetts Medical School, Worcester, MA, United States; ^7^ Department of Biochemistry and Molecular Biology, University of Nebraska Medical Center, Omaha, NE, United States

**Keywords:** tumor-initiating stem-like cells (TICs), extracellular vesicles (EV), mast cells, Alzheimer’s disease, amyloid-beta (Aβ), cell-cell communication, viral hepatitis, steatotic liver disease

The Research Topic “*Cell-To-Cell Communications In Alcohol-Associated, Metabolic-Related And Viral Liver Diseases*” proposed by the journal, Frontiers in Physiology has attracted articles on preclinical and clinical studies that were covered in three review articles and one original research manuscript. Overall, the goal of this topical collection is to provide a better understanding of the molecular mechanism(s) that cause the development and progression of alcohol-associated, metabolic-related, and viral liver diseases. Our hope is that the information in these articles could aid in identifying new biomarkers, therapeutic targets, and promising therapeutic agents that prevent, manage, or reverse disease progression. Here, we, as guest editors, provide a summary of all the articles published in this important issue.

The three review articles in this issue describe the pathogenic mechanism of liver disease progression with a focus on cell-to-cell interaction. A review by Osna et al. discloses the multiple mechanisms through which information exchange occurs between the liver parenchymal and non-parenchymal cells in the context of alcohol-induced end-stage liver disease progression. In particular, the authors highlight the role of extracellular vesicles (EVs), originating from alcohol-exposed liver parenchymal cells (hepatocytes), in activating non-parenchymal cells, including liver macrophages, and hepatic stellate cells. The authors further expound on EV-mediated crosstalk between hepatocytes and liver non-parenchymal cells under HIV and alcohol co-exposure conditions that promotes end-stage liver disease progression. In addition, the authors present information on the crosstalk between cell death pathways and inflammasome activation in alcohol-activated hepatocytes and macrophages. Furthermore, they also provide information on clinical studies that highlight the role of non-inflammatory factors, sinusoidal pressure, and hepatic arterialization, in the pathogenesis of alcohol-induced hepatic fibrogenesis. Importantly, the authors identify therapeutically important cellular/molecular targets that could be used to prevent progressive alcohol-associated liver disease (ALD).

In another review by Machida, the focus is on the tumor-initiating stem-like cell (TIC) population, which are the prime origins of cancer recurrence in drug-treated patients with hepatocellular carcinoma (HCC). This informative review is timely because, while the incidence of many cancers in other tissues is trending downward, that is not applicable to HCC. Therefore, understanding the mechanism(s) of HCC development and its resistance to therapy is important to combat this deadly disease, which has limited treatment options and a dismal 3 year-survival rate of 13–21% in untreated patients. In this review, Machida illustrates the multiple hits by the hepatitis C virus (HCV) that eventually promote transformation and TIC genesis that lead to HCC development. The review article discusses the ten hallmarks of TIC oncogenesis induced by HCV. The author then deliberates on how the understanding of the links between HCV-associated HCC and TIC can aid in providing novel targeting therapy to suppress HCC recurrence and metastasis.

In another review article, Huang et al. explore the role of mast cells in the progression of liver disease. Mast cells play a crucial role in host defense against allergens, parasites, bacteria, and venom. It is known that mast cells upon activation degranulate releasing a variety of mediators (histamine, tryptase, chymase, transforming growth factor-β1, tumor necrosis factor–α, interleukins and other proinflammatory cytokines). Since these factors apparently support the progression of liver disease, Huang et al. skillfully reviews the role of mast cells and their secretory mediators in the pathogenic process during the development of hepatitis, cirrhosis and HCC. In addition, the author discusses the essential role of mast cells in immunotherapy and proposes that targeting mast cell infiltration may be a novel therapeutic option for blocking liver disease progression of diverse etiologies.

An original article by Garcia et al. provides evidence that liver steatosis (fat accumulation), irrespective of etiology, causes impaired clearance of amyloid-beta (Aβ) provoking the development of Alzheimer’s disease (AD). Since heavy alcohol consumption is considered a risk factor for AD, the authors first used an intragastric alcohol feeding mouse model. This alcohol feeding regimen, besides causing massive liver fat accumulation, significantly affect two hepatic proteins, which play important roles in Aβ processing. These two hepatic proteins, lipoprotein receptor-related protein 1(LRP1) and amyloid precursor protein (APP), were also similarly affected in other animal models of ALD (NIAAA chronic-binge alcohol mouse and rat intragastric alcohol feeding models). The authors further prove that the changes in hepatic LRP1 and APP are not alcohol-specific by employing ob/ob mice (mutant mice that obese with significant hepatic steatosis) that also exhibit a decline in LRP1 and higher levels of APP in their livers. Their findings suggest that liver steatosis, rather than alcohol-induced liver injury, is responsible for the regulation of hepatic LRP1 and APP and a likely cause of AD development in the two lifestyle liver diseases, ALD and metabolic-dysfunction associated liver disease (MASLD).

Overall, this topical collection of articles covers important aspects of the molecular mechanism(s) that mediate the development and progression of alcohol-associated, metabolic-related, and viral liver diseases. We recommend the published articles for scientists and physicians involved in basic, translational and/or clinical studies on ALD and MASLD. We strongly feel that the efforts of the guest editors and the journal in developing this topical issue will be a significant step towards understanding, awareness, and hopefully reducing the disease burden.

